# High prevalence of obesity among women in urban Haiti: Findings from a population-based cohort

**DOI:** 10.3389/fpubh.2022.976909

**Published:** 2022-10-05

**Authors:** Eliezer Dade, Miranda Metz, Jean Lookens Pierre, Vanessa Rouzier, Rodney Sufra, Elizabeth Fox, Fabyola Preval, Stephano St-Preux, Jean Ronald Zephir, Wilson Ariste, Rehana Rasul, Shalom Sabwa, Nicholas Roberts, Marie Marcelle Deschamps, Patrice Severe, Daniel Fitzgerald, Jean William Pape, Lily Du Yan, Margaret L. McNairy

**Affiliations:** ^1^Haitian Group for the Study of Kaposi's Sarcoma and Opportunistic Infections (GHESKIO), Port-au-Prince, Haiti; ^2^Center for Global Health, Department of Medicine, Weill Cornell Medicine, New York, NY, United States; ^3^Department of Public and Ecosystem Health, Cornell University, Ithaca, NY, United States; ^4^Institute for Implementation Science in Population Health, City University of New York, New York, NY, United States; ^5^Division of General Internal Medicine, Department of Medicine, Weill Cornell Medicine, New York, NY, United States

**Keywords:** obesity, nutrition transition, cardiovascular disease, food insecurity, overweight, Haiti

## Abstract

**Introduction:**

Obesity is associated with increased risk of non-communicable diseases and death and is increasing rapidly in low- and middle-income countries, including Haiti. There is limited population-based data on body mass index (BMI) and waist circumference (WC) and associated risk factors in Haiti. This study describes BMI and WC, and factors associated with obesity using a population-based cohort from Port-au-Prince.

**Methods:**

Baseline sociodemographic and clinical data were collected from participants in the Haiti CVD Cohort Study between March 2019 and August 2021. Weight was categorized by BMI (kg/m^2^) with obesity defined as ≥30 kg/m^2^. Abdominal obesity was defined using WC cutoffs of ≥80 cm for women and ≥94 cm for men based on WHO guidelines. Sociodemographic and behavioral risk factors, including age, sex, educational attainment, income, smoking status, physical activity, fat/oil use, daily fruit/vegetable consumption, and frequency of fried food intake were assessed for their association with obesity using a Poisson multivariable regression.

**Results:**

Among 2,966 participants, median age was 41 years (IQR: 28–55) and 57.6% were women. Median BMI was 24.0 kg/m^2^ (IQR: 20.9–28.1) and 508 (17.1%) participants were obese. Women represented 89.2% of the population with BMI ≥30 kg/m^2^. A total of 1,167 (68.3%) women had WC ≥80 cm and 144 (11.4%) men had WC ≥94 cm. BMI ≥30 kg/m^2^ was significantly more prevalent among women than men [PR 5.7; 95% CI: (4.3–7.6)], those 40–49 years compared to 18–29 years [PR 3.3; 95% CI: (2.4–4.6)], and those with income >10 USD per day compared to ≤1 USD [PR 1.3; 95% CI: (1.0–1.6)]. There were no significant associations with other health and behavioral risk factors.

**Discussion:**

In Haiti, women have an alarming 6-fold higher obesity prevalence compared to men (26.5 vs. 4.3%) and 89.2% of participants with obesity were women. Abdominal obesity was high, at 44.3%. Haiti faces a paradox of an ongoing national food insecurity crises and a burgeoning obesity epidemic. Individual, social, and environmental drivers of obesity, especially among women, need to be identified.

## Introduction

Overweight and obesity prevalence has nearly tripled globally since 1975, with 39% of adults being overweight and 13% having obesity as of 2016 (1). Obesity is associated with 3.4 million deaths globally each year and an increased risk of diabetes, cancer and cardiovascular disease (CVD). Forty-four percent of the diabetes burden and 23% of the ischemic heart disease burden globally is attributable to overweight or obesity (2, 3). Excessive abdominal fat, as measured by waist circumference (WC), is a stronger predictor of obesity-related health risk than body mass index (BMI) alone, and is associated with Type 2 diabetes, elevated blood pressure, and coronary artery disease. Low- and middle-income countries (LMICs) have the greatest increases in obesity prevalence, and many of these countries face a dual burden of malnutrition and obesity in their populations (4).

The rapid increase of obesity is explained by multiple factors associated with the “nutrition transition” including urbanization, accessibility and affordability of ultra-processed foods high in fat, sugar and salt, and lifestyle factors such as suboptimal dietary practices, as well as lower physical activity (5, 6). Haiti is no exception to the nutrition transition. Haiti has a history of undernutrition and severe food insecurity with up to half of Haitians reporting food insecurity in 2020 (7). At the same time, obesity is rapidly rising. In the last decade, prevalence of obesity among women of reproductive age (15–49 years) in Haiti increased from 6.3% in 2005–2006 to 11.2% in 2016–2017, with higher prevalence among urban compared to rural women (8, 9). The most recent 2016–2017 Demographic and Health Survey (DHS) reported that obesity prevalence defined as BMI ≥30 kg/m^2^ was 23.3% among women and 6.7% among men ages 35–64 (9). Haiti also faces a growing cardiovascular disease (CVD) epidemic, with CVD now the leading cause of death in Haiti, having surpassed HIV and other infectious diseases in the past decade (10).

Reports of obesity prevalence in Haiti vary by study population and few include both BMI and WC distributions. Further, there has not been analysis of the risk factors associated with obesity in the Haitian population, including those specific to urban populations. This is particularly relevant given rapid increases in obesity among young adults globally (2). Getting correct estimates of obesity prevalence based on BMI and WC is essential to know which groups are at highest risk. In this study, we aimed to estimate obesity using BMI and abdominal obesity using WC in the Haiti CVD Cohort Study, a population-based cohort of adults >18 years in Port-au-Prince. We also aimed to identify risk factors associated with obesity in this population.

## Materials and methods

### Study design and population

The study includes participants enrolled in the Haiti CVD Cohort Study, a population-based longitudinal observational study of adults >18 years in Port-au-Prince. Details of study procedures have been previously published (clinicaltrials.gov #NCT03892265) (11). The study was conducted at the Groupe Haitien d'Etude de Sarcome de Kaposi et de Infections Opportunistes, (GHESKIO), a research, treatment, and training clinic located in downtown Port-au-Prince (12). Participants were recruited using multistage random sampling from metropolitan Port-au-Prince which includes an estimated 1.4 million people (13).

Participants were enrolled from March 19, 2019, to August 23, 2021. The analysis in the present study was restricted to participants with information on age, sex, height, weight, and WC (*n* = 2,966, 99% of 3,005 participants enrolled) (Supplementary Figure 1). Pregnant women (*n* = 33) were excluded from analysis.

### Ethics approval and consent to participate

The study protocol and ethical consent forms were approved by Weill Cornell Medicine and GHESKIO institutional review boards (record number 1803019037). Individuals selected for the study provided written informed consent prior to enrollment.

### Measured variables

Measurements at study enrollment include self-reported sociodemographic data (age in years, sex, education, income, occupation) and health behaviors based on standardized WHO STEPS questionnaires (smoking status, physical activity, diet) (14). Age was assessed as a continuous variable and also categorized as 18–29, 30–39, 40–49, 50–59, and 60+ years. Education was categorized as having completed no education, primary, secondary, or higher than secondary school. Daily income was categorized as ≤$1 USD per day, $1–10 USD per day, or >$10 USD per day.

WHO STEPS questions included tobacco use, physical activity, and fruit/vegetable intake (14). Participants were categorized as never smokers or current/former smokers. Physical activity was determined using questions about vigorous activity for longer than 75 min per week, or moderate activity for longer than 150 min per week. Participants were categorized as having low physical activity (no to both questions) vs. moderate to high physical activity (yes to either or both questions). Fruit and vegetable intake was categorized as less than five servings per day or greater than or equal to five servings per day (as per WHO STEPS survey) (14). In addition to WHO STEPS questions, we assessed for fat/oil use and fried food consumption with questions specific to this study suggested by the GHESKIO community advisory board given the Haitian context. Fat/oil use was categorized as rarely/never, sometimes, or often based on self-reported addition of oil, butter, or margarine by the person preparing the foods. Fried food consumption was categorized as 0–1, 2–3, 4–5, or >5 days per week. The dietary questions from the Haiti CVD Cohort Study have been described previously (15).

Clinical data was collected on height (cm), weight (kg), WC (cm). Height and weight were measured using a medical grade mechanical-beam scale with height rod. WC was measured using a medical body tape measurer in centimeters. BMI (kg/m^2^) was categorized for all participants, with underweight defined as <18.5 kg/m^2^, normal weight as 18.5–24.9 kg/m^2^, overweight as 25–29.9 kg/m^2^, and obese as ≥30 kg/m^2^ (14, 16). Cut-offs for abdominal obesity were defined as WC ≥80 cm and ≥94 cm for women and men, respectively, corresponding to WHO classifications (17).

### Statistical analysis

Descriptive statistics were generated using medians for continuous variables and proportions for categorical variables. Participants were categorized as having obesity and abdominal obesity based on WHO cut offs for BMI and WC, respectively. Summary statistics were calculated for the total population and also stratified by populations with obesity and abdominal obesity. Crude prevalence and standard errors (SE) of obesity and abdominal obesity were calculated. Age standardized prevalence was calculated using the WHO 2000–2025 Standard Population with 95% confidence intervals (18).

Distributions of BMI and WC were generated and examined by sex. Correlation between BMI and WC was evaluated using Pearson's correlation coefficients separately for each sex. Proportions of men and women with both obesity and abdominal obesity and those with only abdominal obesity were examined.

Obesity prevalence was stratified by age, sex, education level, income, smoking status, physical activity, occupation, fruit/vegetable intake, and fried food intake categories. Multivariable Poisson regressions were used to investigate whether health behavior and sociodemographic factors were associated with the outcome of obesity for the total population and for men and women separately. For each model, the covariates used were age category, sex, education level, income, smoking status, physical activity, fruit/vegetable intake, fat/oil use, and fried food intake. Robust standard errors were used for all regression models. Adjusted prevalence ratios (PR) and 95% confidence intervals (CI) were reported.

All analyses were performed using R statistical software, version 4.1.3 (19).

## Results

### Population characteristics

A total of 2,966 adults are included in this study. Participants had a median age of 41 years (IQR: 28–55, range: 18–93) and 1,708 (57.6%) were women (Table 1). Among all participants, 1,066 (35.9%) had no education or completed only primary level, and 1,987 (67.0%) earned ≤$1 USD per day. Fat/oil use was high, with 2,540 participants (85.6%) reporting the person cooking their food often added fat/oil. Only 172 participants (5.8%) consumed the recommended 5 servings of fruits and vegetables a day (median two servings per day), and 299 (10.1%) consumed fried food more than 5 days per week. Physical activity varied, with 1,495 (50.6%) of participants reporting low activity.

**Table 1:** Sociodemographic and clinical characteristics for adults in the Haiti CVD Cohort (*N* = 2,966).

	**Total participants**	**Obesity**	**Abdominal obesity**
	***N* (%)**	***N* (%)**	***N* (%)**
**Total sample**	2,966	508	1,311
**Age, years**			
Median [IQR]	41 (28–55)	46 37–56)	46 (35–57)
18–29	869 (29.3)	60 (11.8)	194 (14.8)
30–39	556 (18.7)	105 (20.7)	267 (20.4)
40–49	528 (17.8)	140 (27.6)	307 (23.4)
50–59	499 (16.8)	118 (23.2)	279 (21.3)
60+	514 (17.3)	85 (16.7)	264 (20.1)
**Sex**			
Female	1,708 (57.6)	453 (89.2)	1,167 (89.0)
Male	1,258 (42.4)	55 (10.8)	144 (11.0)
**Education**			
Primary or less	1,066 (35.9)	235 (46.3)	608 (46.4)
Secondary	1,455 (49.2)	224 (44.3)	571 (43.7)
Higher than secondary	439 (14.8)	47 (9.3)	128 (9.8)
**Income (daily)**			
≤1 $USD	1,987 (67.0)	331 (65.2)	868 (66.2)
1–10 $USD	2,435 (15.1)	50 (9.8)	174 (13.2)
>10 $USD	525 (17.7)	125 (24.6)	265 (20.2)
**Smoking status**			
Never	2,714 (91.8)	476 (94.1)	1,225 (93.8)
Current/former	243 (8.2)	30 (5.9)	81 (6.2)
**Physical activity**			
≤ 150 min / week (low)	1,495 (50.6)	250 (49.5)	676 (51.8)
>150 min / week (moderate-high)	1,460 (49.4)	255 (50.5)	630 (48.2)
**Alcohol use**			
<1 drink a day (low)	2,842 (96.3)	493 (98.0)	1,277 (98.0)
≥1 drink a day (moderate-high)	110 (3.7)	10 (2.0)	26 (2.0)
**Fruit/vegetable intake**			
Median daily serving [IQR]	2.0 [1.0, 2.0]	2.0 [1.0, 2.0]	2.00 [1.00, 2.0]
<5 servings a day	2,786 (94.2)	470 (92.9)	1,225 (93.8)
≥5 servings a day	172 (5.8)	36 (7.1)	81 (6.2)
**Oil use**			
Often	2,540 (85.6)	416 (81.9)	1,075 (82.0)
Sometimes	301 (10.1)	64 (12.6)	162 (12.4)
Rarely/Never	119 (4.0)	26 (5.1)	70 (5.3)
**Fried food intake per week**			
0–1 day	1,813 (61.3)	344 (68.0)	860 (65.8)
2–3 days	682 (23.0)	107 (21.1)	277 (21.2)
4–5 days	166 (5.6)	18 (3.6)	60 (4.6)
>5 days	299 10.1)	37 (7.3)	110 (8.4)

IQR, interquartile range; min, minute.

The estimated age-standardized prevalence of obesity using BMI categories was 17.2% (95% CI 16.4–17.9%). Obese participants with BMI ≥30 kg/m^2^ (*n* = 508) had a median age of 46 years (IQR: 37–56), and 89.2% were women. Low education was common, with 235 participants with obesity (46.2%) having completed a primary education or less, and 331 of participants with obesity (65.1%) earning ≤$1 USD per day. Fat/oil use was high, with 416 of participants who were obese (81.9%) reporting often addition of fat/oil to food, and 36 (7.1%) participants who were obese consumed at least 5 servings of fruits and vegetables per day.

A total of 1,311 participants (44.3% age standardized, 95% CI 43.1–45.5%) were categorized as having abdominal obesity using WC. The median age of participants with abdominal obesity was 46.0 (IQR: 35.0–57.0), and 89.0% were women. Among participants with abdominal obesity, 608 (46.4%) had completed a primary education or less, and 868 (66.2%) earned ≤$1 USD per day. Among participants with abdominal obesity, proportions of smoking and physical activity were similar compared to the general study population, along with fruit/vegetable and fried food intake.

### BMI and WC distribution by sex

**Figure 1:**
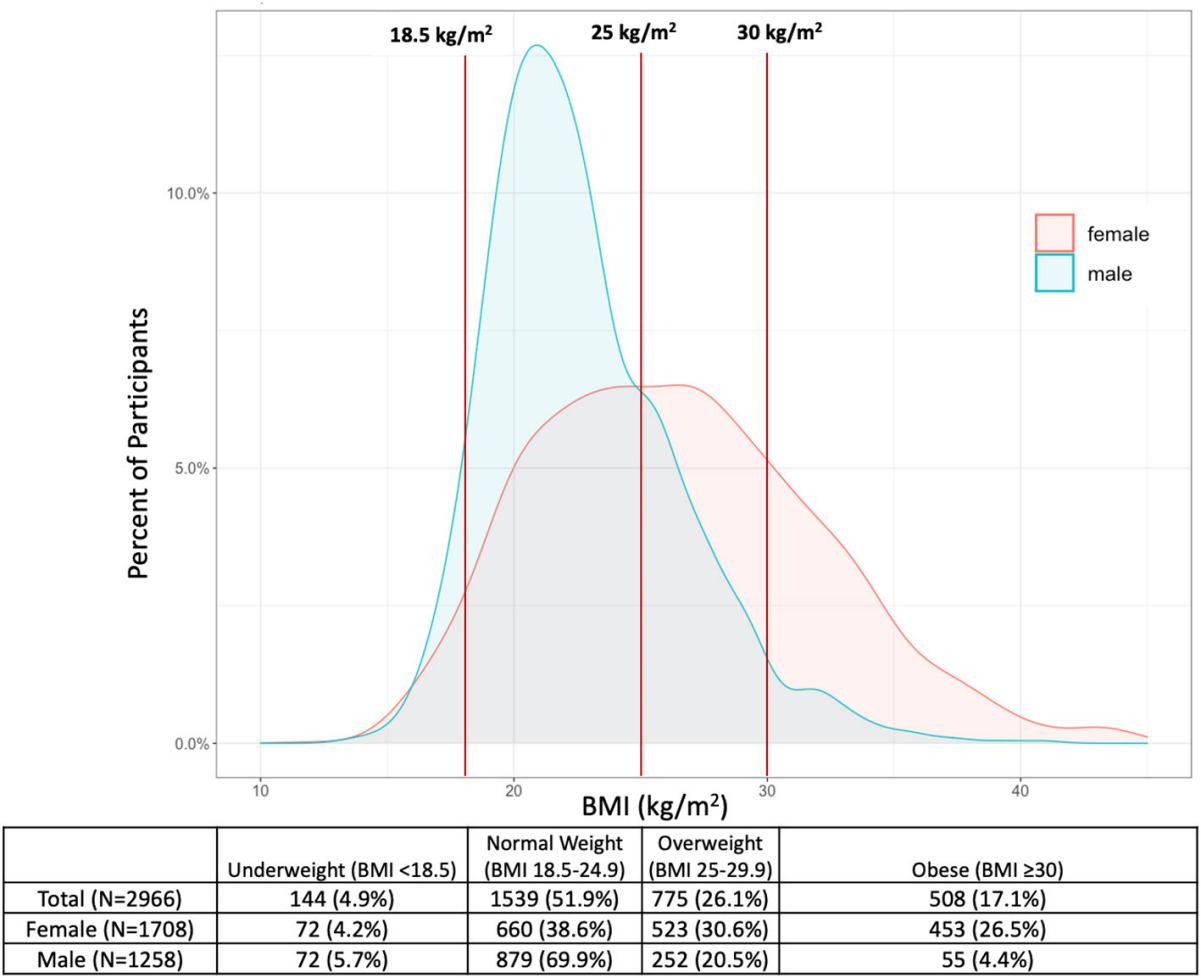
Distribution of BMI among male and female adults in the Haiti CVD Cohort (*N* = 2,966). Vertical lines represent cutoffs for WHO BMI categories at 18.5, 25, and 30 kg/m^2^.

**Figure 2:**
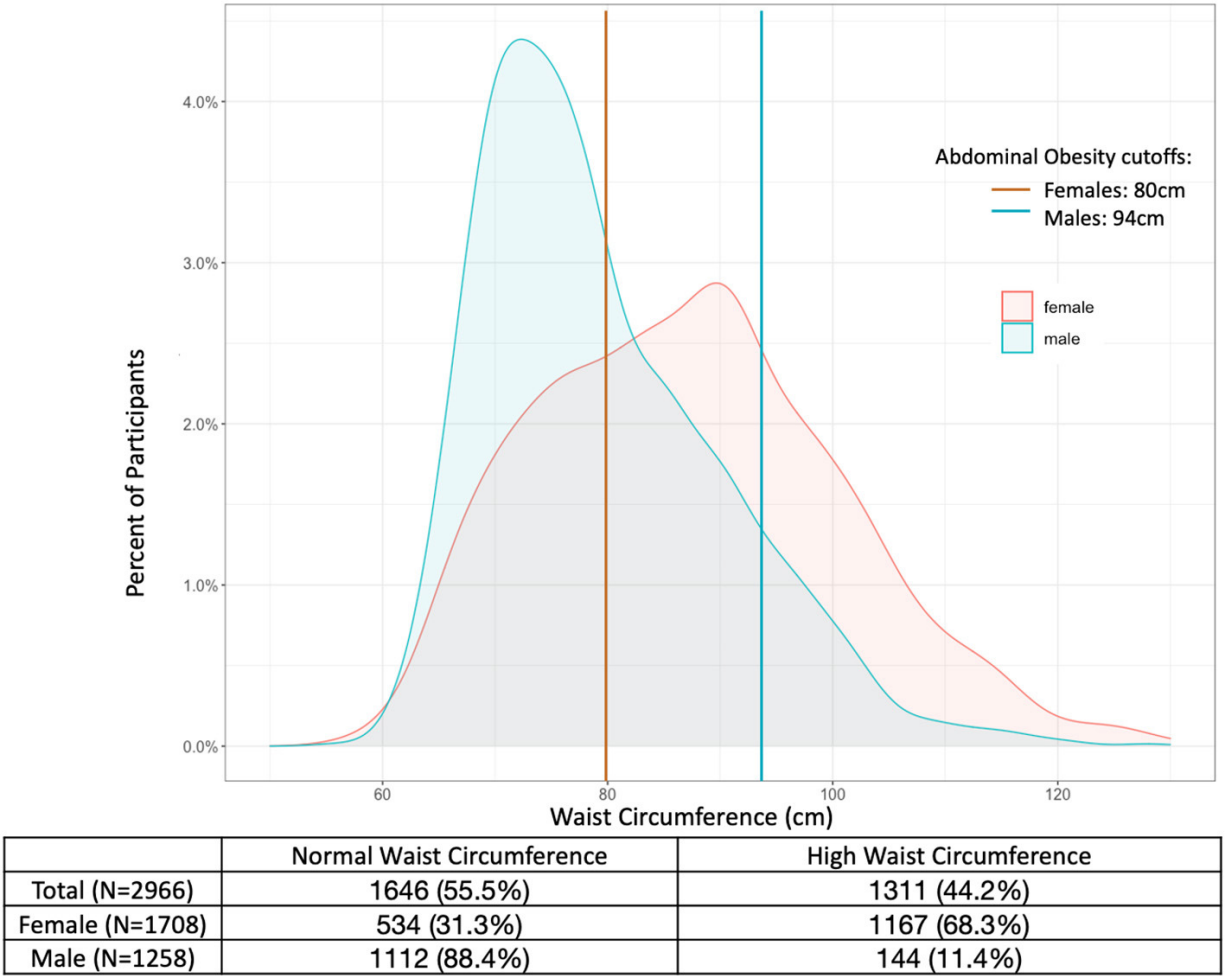
Distribution of waist circumference among male and female adults in the Haiti CVD Cohort (*N* = 2,966). Lines represent cutoffs for abdominal obesity at 80 cm for females (red) and 94 cm for males (blue).

The median BMI for all participants was 24.0 kg/m^2^ (IQR: 20.9–28.1), with 144 participants (4.9%) categorized as underweight, 1,539 (51.9%) categorized as normal weight, 775 (26.1%) as overweight, and 508 (17.1%) as obese. The median BMI for women was 26.2 kg/m^2^ (IQR: 20.1–30.3), and the median BMI for men was 22.0 kg/m^2^ (IQR: 20.1–25.0), with 453 women (26.5%) and 55 men (4.4%) categorized as obese (Figure 1). The median WC was 87.0 cm for women (IQR: 77.0–96.0) and 77.0 cm for men (IQR: 71.0–86.0). Among women, 1,167 (68.3%) had WC ≥80 cm, while 144 men (11.4%) had WC ≥94 cm (Figure 2).

The correlation between BMI and WC was 0.88 for women and 0.82 for men. Among women, 451 (26.4%) had WC ≥80 cm and BMI ≥30 kg/m^2^ (both obesity and abdominal obesity) compared to 50 (4.0%) of men who had WC ≥94 cm and BMI ≥30 kg/m^2^. Among women, 716 (41.9%) had WC ≥80 cm but BMI <30 kg/m^2^ (abdominal obesity only); among men, 94 (7.5%) had WC ≥94 cm but BMI <30 kg/m^2^.

### Obesity prevalence across sociodemographic groups and factors associated with obesity

**Figure 3:**
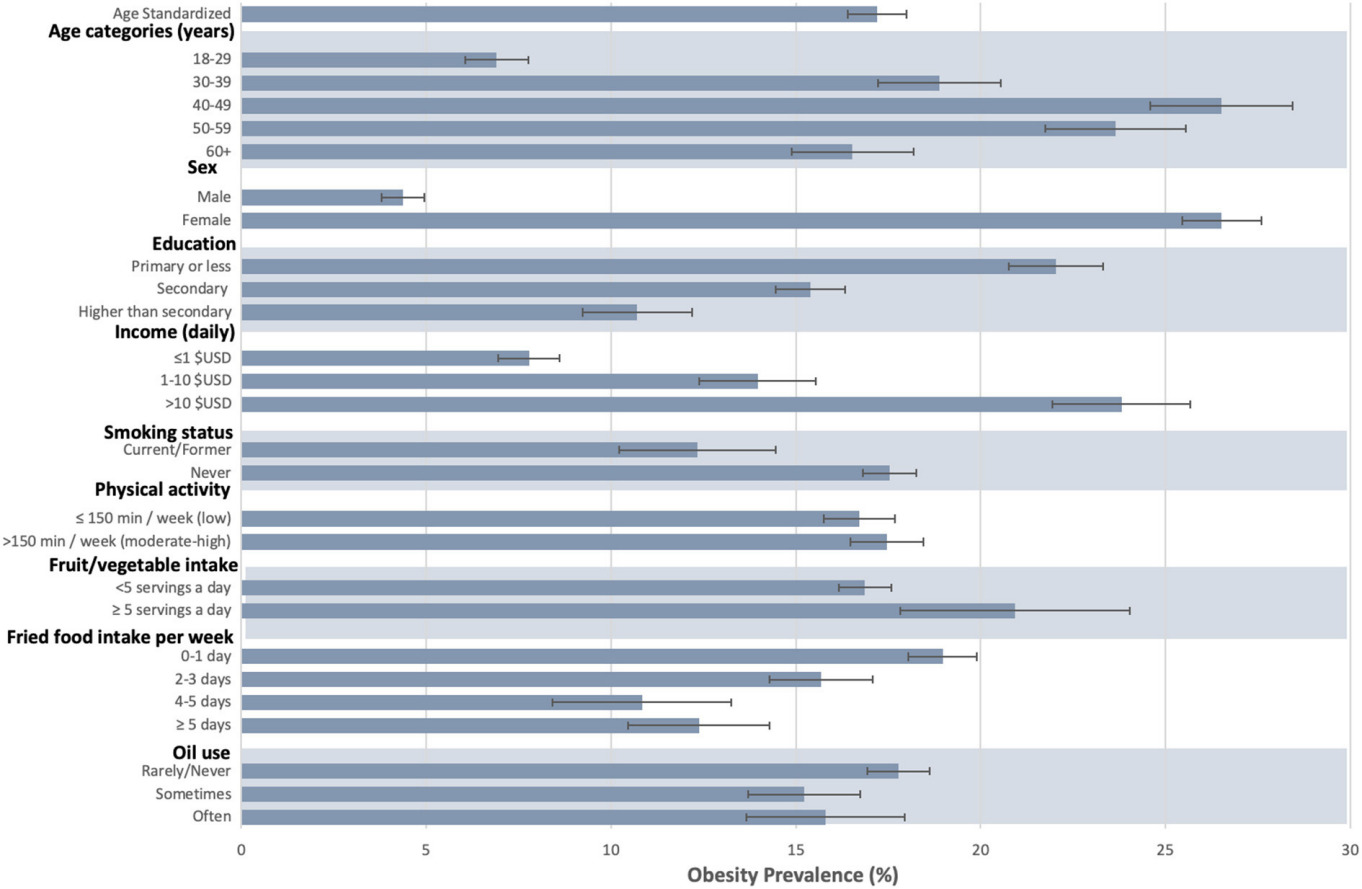
Prevalence of obesity by sociodemographic factors and heath behaviors among adults in the Haiti CVD COHORT (*N* = 2,966). Gray bars represent obesity prevalence by group, and black lines the standard error.

Figure 3 describes the prevalence of BMI ≥30 kg/m^2^ among adults in the entire population and across sociodemographic groups. Women and participants aged 40–49 had the highest prevalence of obesity, with 26.5% (95% CI 25.5–27.6%) of women and 26.5% (95% CI 24.6–28.4%) of participants aged 40–49 categorized as obese. Participants who earned >$10 USD per day had a higher prevalence of obesity (23.8%, 95% CI 21.9–25.7), compared to those who earned ≤$1 USD per day (7.7%, 95% CI 6.9–8.6%). Participants with primary or less education had an obesity prevalence of 22.0% (95% CI 20.8–23.3), compared to 10.7% (95% CI 9.2–12.2) of those with higher than secondary education.

Factors associated with obesity ≥30 kg/m^2^ were assessed (Table 2). Female sex was associated with increased obesity prevalence compared to male sex [PR 5.7; 95% CI: (4.3–7.6)]. Age was also associated with obesity, with individuals aged 40–49 years 3.3 times more likely [95% CI: (2.4–4.6)] to have obesity compared to participants aged 18–29 years. Participants who earned >$10 USD per day were more likely to have obesity (PR 1.3, 95% CI: 95% CI 1.0–1.6) compared to those who earned ≤$1 USD per day. There were no statistically significant associations between obesity and smoking status [current/former smoker vs. never smoker PR 1.0 95% CI: (0.6–1.3)], physical activity [low physical activity vs. high physical activity PR 1.1 95% CI: (0.9–1.3)], fat/oil use [rarely/never vs. often PR 1.0 95% CI: (0.6–1.4)], fruit and vegetable intake [≥5 servings/day vs. <5 PR 1.1 95% CI: (0.8–1.6)], or fried food intake [>5 days vs. 0–1 day PR 0.9 95% CI: (0.6-1.3)].

**Table 2:** Prevalence ratios (PR) of obesity (BMI > 30 kg/m^2^) among adults in the Haiti CVD Cohort using multivariable poisson regressions for the total population, women, and males.

	**Total population (*N* = 2,966)**	**Males (*N* = 1,258)**	**Females (*N* = 1,708)**
**Age categories (years)**	**Obesity PR [95% CI]**	**Obesity PR [95% CI]**	**Obesity PR [95% CI]**
18–29	Ref	Ref	Ref
30–39	**2.3 [1.7**–3.3]^*^	1.8 [0.6–5.1]	**2.3 [1.6**–3.3]^*^
40–49	**3.3 [2.4**–4.6]^*^	**6.5 [2.8**–16.4]^*^	**2.9 [2.0**–4.1]^*^
50–59	**3.1 [2.2**–4.4]^*^	**4.4 [1.6**–12.3]^*^	**2.8 [1.9**–4.1]^*^
60+	**2.5 [1.7**–3.6]^*^	**5.2 [1.7**–15.5]^*^	**2.2 [1.5**–3.4]^*^
**Sex**			
Male	Ref		
Female	**5.7 [4.3**–7.6]^*^		
**Education**			
Primary or less	Ref	Ref	Ref
Secondary	1.1 [0.9–1.3]	2.0 [0.9–4.6]	1.0 [0.8–1.3]
Higher than secondary	1.1 [0.8–1.5]	**3.8 [1.5**–10]^*^	0.9 [0.6–1.3]
**Income (daily)**			
≤ 1 USD	Ref	Ref	Ref
1–10 USD	0.8 [0.6–1]	1.2 [0.5–2.4]	**0.7 [0.5**–1.0]^*^
>10 USD	**1.3 [1.0**–1.6]^*^	1.1 [0.5–2.3]	**1.3 [1.0**–1.6]^*^
**Smoking status**			
Never	Ref	Ref	Ref
Current/former	0.9 [0.6–1.3]	0.8 [0.3–1.8]	1.0 [0.6–1.4]
**Physical activity**			
>150 min / week (moderate-high)	Ref	Ref	Ref
≤ 150 min / week (low)	1.1 [0.9–1.3]	0.9 [0.5–1.6]	0.9 [0.7–1.1]
**Oil Use**			
Often (ref)	Ref	Ref	Ref
Sometimes	0.9 [0.7–1.2]	0.4 [0.1–1.4]	1.0 [0.7–1.3]
Rarely/never	1.0 [0.6–1.4]	0.4 [0–1.9]	1.0 [0.6–1.5]
**Fruit/vegetable intake**			
<5 servings a day	Ref	Ref	Ref
≥ 5 servings a day	1.1 [0.8–1.6]	1.3 [0.3–3.8]	1.1 [0.8–1.6]
**Fried food intake per week**			
0–1 day	Ref	Ref	Ref
2–3 days	0.9 [0.7–1.1]	0.6 [0.2–1.3]	0.9 [0.7–1.2]
4–5 days	0.7 [0.4–1.1]	0.9 [0.2–2.6]	0.7 [0.4–1.1]
>5 days	0.9 [0.6–1.3]	1.1 [0.4–2.5]	0.9 [0.6–1.3]

PR, prevalence ratio; CI, confidence interval.

Bold values with

* represent statistically significant PR with 95% CI.

In sex-stratified analysis, men had stronger associations between increased age and obesity prevalence, with men 40–49 years being 6.5 times likelier (95% CI 2.8–16.4) to have obesity compared to men aged 18–29 years, while women aged 40–49 years were 2.9 times likelier (95% CI 2.0–4.1) to have obesity compared to women aged 18–29 years. Men who had higher than secondary education were significantly more likely to have obesity [PR 3.8, 95% CI: (1.5–10.0)], while women had no association between obesity and education. Compared to those that earned ≤$1 USD per day, women who earned $1–10 USD were less likely to have obesity (PR 0.7, 95%CI 0.5–1.0), while those who earned >$10 USD per day were more likely to have obesity (PR 1.3, 95% CI: 95% CI 1.0–1.6). Among men, there was no association between income and obesity prevalence. None of the other sociodemographic or behavioral factors were significantly associated with obesity for men or women.

## Discussion

The age-standardized prevalence of obesity defined by BMI ≥30 kg/m^2^ was 17.2% in this population-based cohort of adults in Port-au-Prince. The rate of abdominal obesity was even higher, at 44.3%. Obesity prevalence was 6-fold higher in women compared to men (26.5 vs. 4.4% among men), with 89.2% of those with obesity were women. Higher income was associated with BMI ≥30 kg/m^2^; however, other common risk factors, namely, poor diet and physical inactivity, were not significantly associated with increased risk of obesity in our study population.

Our data point to high prevalence of obesity in Haiti, similar to prior estimates from the 2016–2017 DHS, where 23.3% of women and 6.7% of men aged 35–64 were obese (9). Compared to other LMICs, the obesity prevalence in our study population is lower among men but higher among women (20). Our finding that women have a higher prevalence of obesity is consistent with other studies in the Caribbean, and around the world. Yet the magnitude of sex differences between women and men in our study is larger than prior studies (21–24). A meta-analysis of 27 studies in the Caribbean found that women are 3.1 times (95% CI: 2.4–3.9) more likely to have obesity than men (23), and our study found a 6-fold difference. The large gap in obesity prevalence by sex is likely due to a confluence of risk factors unique to women, including lower status, diet patterns, child bearing history, gender inequality, and cultural preferences about body size and shape (21–23, 25, 26). In many LMICs, including the Caribbean, obesity among women has historically been associated with wealth and perceived as a sign of strength and good health (26–29). In South Africa, weight loss is associated with sickness such as HIV and TB and often stigmatized among black women (30, 31). These views are often held by women from low-socioeconomic status categories (32). Based on observational data, these beliefs seem to be held in Haiti as well; larger body size among Haitian women is socially accepted. Haitian women want to appear overweight or obese because it is a sign of good health, particularly for HIV patients. Obesity could additionally be seen as a sign of social and economic success in areas with extreme poverty. Given the higher prevalence of obesity among women than men at all age categories, the underlying drivers of the sex gap may occur before adulthood and thus are not captured in our survey. Social inequalities starting in childhood may lead to higher obesity among women at all ages, as women may be expected to do different tasks and be more likely to engage in sedentary activity in the home (25). Regardless the cause of these sex-differences in obesity, the extremely high rates of obesity in women is a major public health concern given the association of obesity and CVD risks (2, 3), as well as intergenerational impacts of obesity of women of reproductive age (33, 34).

Our study adds to the literature by including WC measurements to estimate abdominal obesity. While BMI is traditionally used to measure obesity and has been independently associated with increased CVD risk factors and events (4, 11), abdominal obesity has been linked to higher mortality independent of BMI (35), and myocardial infarction has been shown to be more strongly associated with abdominal obesity than with BMI (36). Our finding that 41.9% of women and 7.5% of men have high WC but BMI <30 kg/m^2^ suggests that at-risk adults, and particularly women, may be missed in clinical settings if BMI alone is used as a cutoff. Indeed, current estimates of obesity are likely underestimating the actual burden given national surveys in Haiti typically only report obesity by BMI (9). Adjustments to clinical guidelines for obesity diagnoses might benefit from inclusion of WC.

We also found a higher prevalence of obesity among middle-aged and higher-income adults. Our findings align with global analyses which demonstrate peak obesity during 45–55 years (2). Middle-aged adults may be less physical active, which in Haiti might be exacerbated by recent political violence and social unrest which precludes safe outdoor spaces for exercise and has increased the use of motorcycles over walking as the main form of transport. Middle aged persons may also be more financially stable than younger and older age groups, which aligns with our findings that higher income was associated with higher prevalence of obesity. This is consistent with existing literature across LMICs (21, 37, 38). A study evaluating obesity prevalence using DHS data from 49 LMICs found that overweight/obesity increased with increasing socioeconomic status in 44 countries (39, 40). This may be due to “westernization” of diets to higher fats and processed foods among persons with increasing income (38, 41). In LMICs like Haiti, it may only be the wealthy who can afford to buy sufficient quantities and calories of food that lead to obesity (38). Despite this finding of higher income associated with modest increase in obesity, it is important to note that Haiti is one of the most food insecure countries in the world, with nearly 50% of the population in need of food assistance (42). More local research is needed to understand the relationship between income, food insecurity, and obesity in LMICs and may reveal multiple pathways leading to obesity (43–46).

Strengths of this study include the use of a large population-based cohort data among populations in informal settlements in Haiti and the use of two clinical objective measures of obesity, BMI and WC. WC cutoffs for obesity are not specific for Caribbean-specific population. Self-reported diet, physical inactivity and other health behavioral data may result in recall bias. Further, this cohort was exclusively from urban Port-au-Prince, so study findings may not be directly comparable with obesity studies in rural Haiti or other LMICs.

## Conclusion

In summary, we report an age-standardized obesity prevalence of 17.2%, and an abdominal obesity prevalence of 44.3% in this population-based cohort of adults in Port-au-Prince. Women account for the vast majority of adults who are obese and the gender gap in obesity is larger than reported in other LMICs. Understanding individual, social and environmental drivers of obesity, especially among women, in an extremely food insecure country such as Haiti are needed to implement targeted interventions. In an under resourced country with fragile health infrastructure, there will be immense health consequences for the population if the obesity epidemic is not addressed.

## Data availability statement

The raw data supporting the conclusions of this article will be made available by the authors, without undue reservation.

## Ethics statement

The studies involving human participants were reviewed and approved by Weill Cornell Medicine GHESKIO Institutional Review Boards. The patients/participants provided their written informed consent to participate in this study.

## Author contributions

ED, MM, and MLM: conceived study. ED, JLP, RS, VR, FP, SS-P, and WA: data curation. ED and MM: formal analysis and writing—original draft preparation. MLM: funding acquisition. RS, JLP, ED, and VR: investigation. ED, EF, MM, MLM, and LY: methodology. ED, JLP, RS, VR, MD, and JWP: project administration and resources. MM: software. ED, JLP, RS, VR, MM, SS, FP, MD, NR, JWP, MLM, and LY: writing—review and editing. All authors have read and confirm that they meet the criteria for authorship, contributed to the article, and approved the submitted version.

## Funding

ED, RS, JLP, VR, RR, JWP, and MLM report a grant from NHLBI R01HL143788. MLM reports a grant from NHLBI D43TW011972 and Fogarty International Center R21TW011693.

## Conflict of interest

The authors declare that the research was conducted in the absence of any commercial or financial relationships that could be construed as a potential conflict of interest.

## Publisher's note

All claims expressed in this article are solely those of the authors and do not necessarily represent those of their affiliated organizations, or those of the publisher, the editors and the reviewers. Any product that may be evaluated in this article, or claim that may be made by its manufacturer, is not guaranteed or endorsed by the publisher.

## Supplementary material

Supplementary Figure 1 Inclusion and exclusion criteria for final analytic dataset.
