# Supplementary figures and images for: High prevalence of obesity among women in urban Haiti: Findings from a population-based cohort
Source: Front Public Health. 2022 Oct 5;10:976909. doi: 10.3389/fpubh.2022.976909 (PMC9581236; doi:10.3389/fpubh.2022.976909)

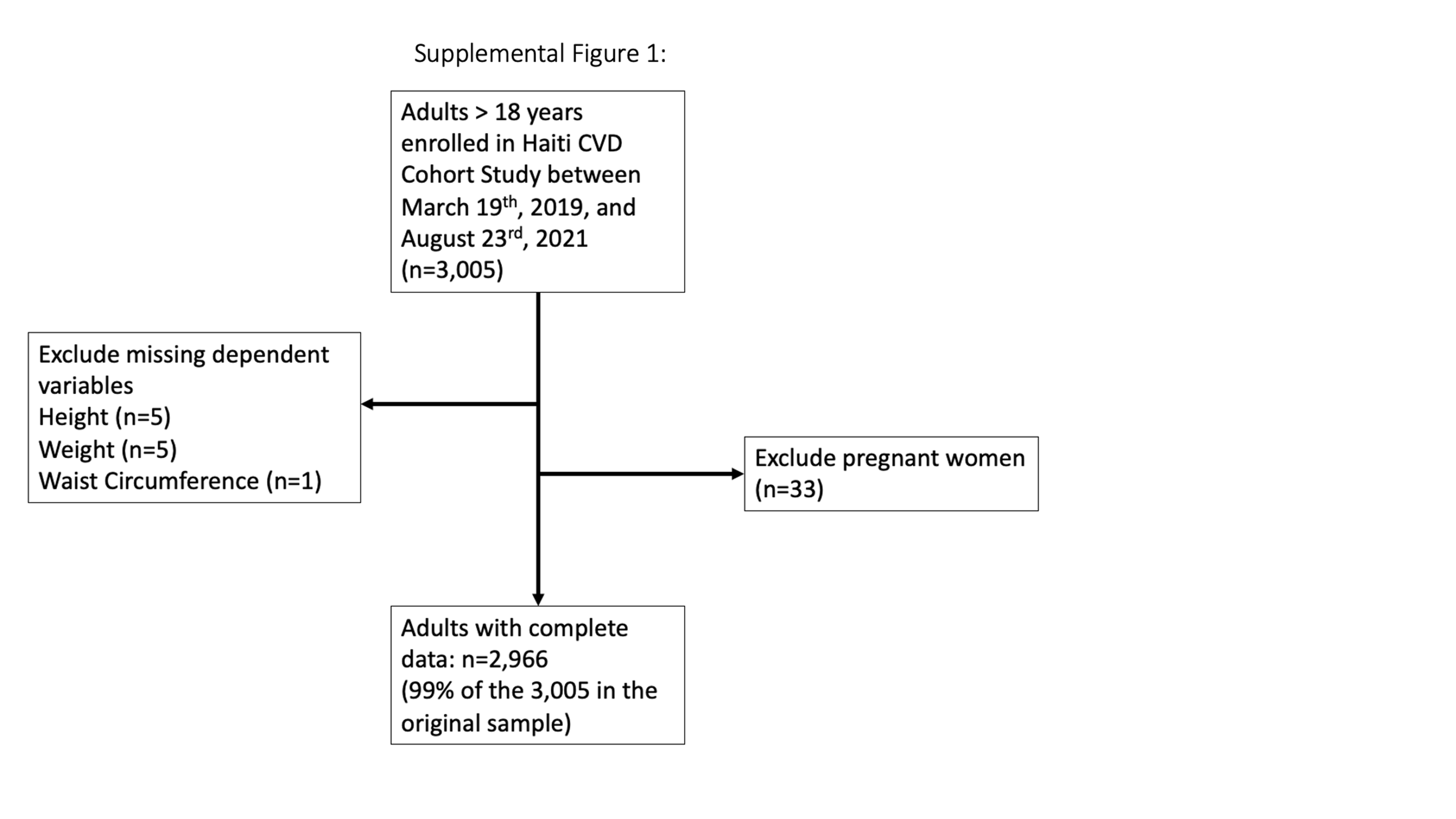

Supplement: Supplementary Figure 1 — Inclusion and exclusion criteria for final analytic dataset. [file Image_1.TIFF]
